# Investigation of the Mozart sonata effect on contextual memory extinction

**DOI:** 10.31744/einstein_journal/2025AO1299

**Published:** 2025-07-02

**Authors:** Rodolfo Souza de Faria, Amanda Di Gesu, Lelis do Vale Miranda, Luciano Magalhães Vitorino, Clarissa Maria Ferreira Trzesniak, Cesar Renato Sartori

**Affiliations:** 1 Faculdade de Medicina de Itajubá Itajubá MG Brazil Faculdade de Medicina de Itajubá, Itajubá, MG, Brazil.; 2 Universidade Estadual de Campinas Campinas SP Brazil Universidade Estadual de Campinas, Campinas, SP, Brazil.

**Keywords:** Fear, Memory, Music, Amnesia, retrograde, Mice

## Abstract

**Objective:**

This study aimed to analyze the influence of Mozart’s Sonata K.448 on contextual memory extinction in male mice.

**Methods:**

Male mice were divided into three groups (G1-Mozart, G2-Ambience, G3-Control). Throughout the project, only the first group was exposed to the sonata at the intrauterine stage. Only Groups 1 and 2 underwent aversive training; subsequently, all groups participated in extinction and recall tests.

**Results:**

On the first day of extinction, G1’s freezing time was longer than G2’s and G3’s, whilst on the second day it was only longer than G3’s. Finally, on the fifth day, G1’s freezing time was shorter than that of G2. During the second and third days of extinction, G1’s freezing time decreased more than G3’s, whereas during the fourth and fifth day, G1 upheld its freezing time, whereas G2’s extended. During recall, G1 had a longer freezing time than G3. All results were statistically significant (p≤0.05).

**Conclusion:**

Based on the results, we concluded that male mice exposed to Mozart’s Sonata K.448 showed increased freezing time, suggesting an impact of the music on memory formation. However, they exhibited a linear extinction memory process. This progressive decrease in freezing behavior was not observed in the ambient and Control Groups.

## In Brief

This study analyzed the influence of Mozart’s Sonata K.448 on contextual memory extinction in male mice. The mice were divided into three groups, with only G1 mice exposed to the sonata. G1 initially showed longer freezing times but exhibited a linear extinction process, unlike G2 and G3. These results suggest that the sonata affected memory formation while aiding progressive extinction.

## Highlights

■ In this study, mice were exposed to music at the intrauterine stage.

■ Music exposure before aversive memory formation enhances neuroplasticity, aiding in extinction.

■ The recall test showed that music exposure had sustained beneficial long-term effects.

## Graphical abstract

**Figure:**
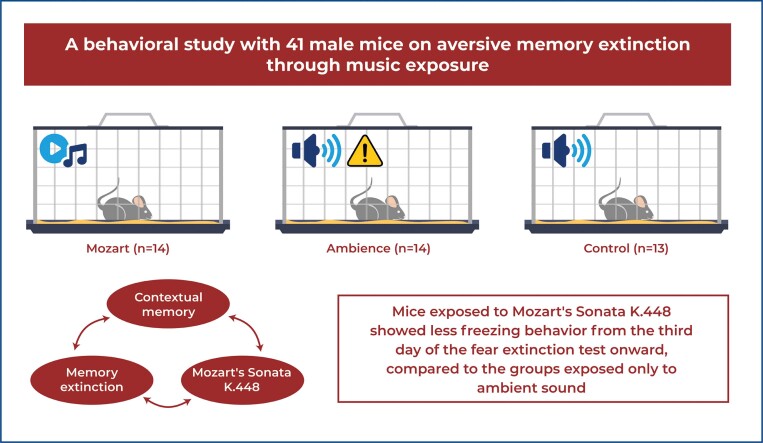

## INTRODUCTION

Memory refers to the ability to store and recall information acquired through experience.^1,2^ Memory can be classified according to its duration into three types: immediate, short-term memory and long-term memory (LTM). It can also be classified according to its content into declarative or explicit memory and procedural memory. Immediate memory refers to the brief retention of information by the prefrontal cortex. Short-term memory is an organized form of memory that lasts for a few hours. In contrast, LTM can last a lifetime because it involves changes in synapses and is retained in the hippocampus through protein synthesis and brain-derived neurotrophic factor (BDNF) signaling. Declarative or explicit memory relates to conscious recall of retained information, whereas procedural memory is unconsciously evoked.^2-4^

The formation of memory in the brain occurs in sequential stages including encoding, consolidation, storage, and activation.^3^ Encoding involves the comprehension and integration of information into a neural network. Consolidation is the phase during which new information is associated with old information, and storage is configured when the memory circuitry is repeatedly activated to preserve it. Activation refers to the retrieval of information, that is, the process of transferring it to consciousness for use in active cognition. Once consolidated, the memory can be maintained (as in LTM) in cases of repeated recall or extinguished.^3,4^

Memory research has been conducted in various animal models; however, as mice share a similar behavioral basis with humans and are easily handled, they are the standard animal models for study development.^5,6^ In this case, memory evaluation was performed by analyzing behavioral changes.^1^ In the early 20th century, Pavlov demonstrated that for the extinction of memory, the conditioned and unconditioned stimuli must be unlinked to eliminate the previously learned response.^7^ The conditioned stimulus, represented by the context to which the mice were exposed (cage with lighting and sound bursts), was associated with the unconditioned stimulus (electric shock), generating a contextual fear memory manifested through freezing behavior in mice. To erase the learned contextual memory, the mice were exposed solely to the conditioned stimulus without the accompanying shock, thereby dissociating it from the context and suppressing the physical fear memory of the shock.^7-9^

Therefore, the extinction of memory has great adaptive value, as it prevents us from persisting in behaviors or maintaining thoughts that are no longer connected to reality. In psychotherapy, exposure therapy is based precisely on the extinction of memories through the repetition of stimuli relevant to traumatic memories but without trauma.^7,10-12^

In the brain, music triggers changes in the modulation of neural circuits, gene expression, neuronal activity, and neurotrophin production [an increase in BDNF and a decrease in nerve growth factor (NGF)], thus favoring memory formation. Music has also been shown to influence the physiological responses to emotions in the mesolimbic area.^13-15^ Emotions are an important factor in memory consolidation. During memory formation, the amygdala increases neuronal activity by hormonal release, favoring synaptic plasticity and cortico-hippocampal interactions.^2-4^ Yet not all musical stimuli favor cognitive function.^13^ Evidence suggests that stimulant music enhances arousal and boosts memory performance.^16^ Rauscher suggested that the complexity of the musical composition would define the animal model performance by comparing outcomes from mice exposed to different classical pieces.^14^ Aoun evidenced that suggestion by proving white noise and minimalist piano wouldn’t enhance mice behavior.^17^ However, new theories, tested in humans, demonstrated that the music type, perception, and preference of the individual are decisive factors in the stimuli influence, such that unpleasant or over-stimulating sounds might even worsen the performance.^16^

In research exploring the connection between the brain and music, Mozart’s Sonata K.448 is frequently employed due to its intricate composition and the findings that, while still not fully understood, highlight its effects. Animals exposed to this sonata show a significant, albeit temporary (10 to 15 min), improvement in tasks requiring spatial and temporal memory through neuroplasticity and its impact on cortical and cerebellar areas. The Mozart Effect refers to the potential to enhance cognitive function, and its generalization has been carried out in rodents to eliminate the social and personal components present in human research.^13,15-17^

However, the literature lacks studies related to the effects of music on brain modulation and the consequent extinction of contextual memory. A search was conducted using the descriptors “memory extinction and K. 448,” “memory extinction and music,” and “memory extinction and brain modulation and music” in relevant databases, such as PubMed, and only four articles were found. Therefore, the present research aims to experiment with and find evidence of the relationship between Mozart’s Sonata K.448 and the extinction of contextual memory.^18-21^

## OBJECTIVE

This study aimed to analyze the influence of intrauterine exposure to Mozart’s Sonata K.448 on fear-memory extinction in mice. This study investigated whether listening to classical music facilitates the extinction of contextual memory. These findings may have beneficial implications for the treatment of anxiety disorders in clinical practice.

## METHODS

### Animals

In the present study, 12 pregnant female C57BL/6 mice aged 3-4 months were obtained from the animal facility at the *Faculdade de Medicina de Itajubá*. After birth, all male offspring were separated and randomly divided into three groups: G1, Mozart (n=14); G2, Ambience (n=14); and G3, Control (n=13).

The sample size was based on standard practices observed in similar behavioral studies and prior experimental designs, which consistently yielded sufficient statistical power to detect significant effects when examining behavioral differences in mice.^11,13-15,17-19^

Male mice were selected based on potential behavioral differences influenced by female sex hormones, particularly progesterone.^22^ The animals had free access to water and Purina^®^ commercial chow “ad libitum” and were kept in plastic cages on a 12-h light-dark cycle with five animals from the same group per cage.

The same animals were used in the project “Investigation on the Relationship Between Mozart’s Sonata K.448 and Sound Memory Extinction,” also under the guidance of Prof. Dr. Rodolfo Souza de Faria, at the Laboratory of Memory Neurophysiology (LNM). The procedures were approved by the Animal Use Ethics Committee (CEUA) of the *Faculdade de Medicina de Itajubá* (FMIt) and registered under No. 10/05/2018.

### Behavioral procedures

**Figure 1:**
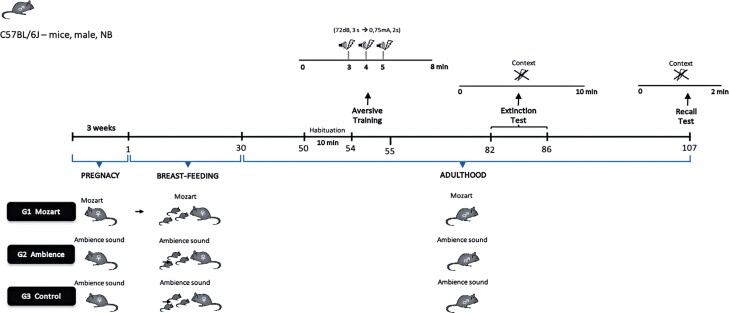
Experimental procedures

The behavioral procedures illustrated in figure 1 are based on the work of Matsuda et al., which is a consolidated method in the literature, in addition to the appliances available at LNM.^23^

The first group was exposed to Mozart’s Sonata K.448, whereas the second and third groups were not. The Ambience Group underwent the same behavioral procedures as the Mozart Group. The Control Group was not subjected to aversive training. In this way, it was possible to determine not only the influence of musical exposure but also the effectiveness of the extinction protocol and the difference in the behavior of mice not exposed to trauma.

### Music exposure during gestation

Initially, 12 pregnant female C57BL/6 mice aged 3-4 months were used in the study. The groups were exposed to their respective sounds (Sonata K. 448 and ambient sounds) from mating until birth. They were divided into three groups: G1, Mozart, exposed to Mozart’s Sonata K.448 (n=4); G2, ambience, exposed to ambient sound (n=4); G3, control, exposed to ambient sound (n=4). The selected females were maintained in individual cages and received the corresponding sound stimuli. G1 was exposed to the sonata at an average intensity of 60-70 dB for 10 h a day from 9:00 PM to 7:00 AM throughout the gestation period.^15^

### Music exposure during lactation

After birth, the offspring were kept in individual cages with their respective mothers according to the three groups. The groups were exposed to their respective sounds (Sonata K. 448 and ambient sounds) during lactation. Group G1 - Mozart: exposed to Mozart’s Sonata K.448 (n=4 females + offspring); G2 - Ambience: exposed to ambient sounds (n=4 females + offspring); G3, Control: exposed to ambient sounds (n=4 females + offspring). G1 received the corresponding sound stimulus at an average intensity of 60-70 dB for 10 h per day from 9:00 PM to 7:00 AM throughout the lactation period.^15^

### Music exposure in adulthood

Male mice from each litter were separated from their mothers after lactation (30 days). Fifteen male offspring were randomly selected from each group. These mice were exposed from the 30th day to the 107th day to the same music given to the mother during mating, gestation, and lactation. They were divided into the following groups: G1, Mozart, exposed to Mozart’s Sonata K.448 (n=14); G2, ambience, exposed to ambient sound (n=14); G3, Control, exposed to ambient sound (n=13). Each group was allocated to three cages, with only five males from the same group in each cage. G1 received the corresponding sound stimulus at an average intensity of 60-70 dB for 10 h per day from 9:00 PM to 7:00 AM.^15^

### Habituation

After 50 days of music exposure in adulthood, mice from all groups (G1, G2, and G3) underwent a 5-day habituation period (50th, 51st, 52nd, 53rd, and 54th days), during which each mouse spent 10 min in the conditioning chamber. This procedure aimed to control behavioral biases related to the novelty of the ambience to which the mice were exposed during the aversive training session. The chambers were cleaned with 70% ethanol before and after each treatment.^23^

### Aversive training

On the 55th day, mice from groups G1 (Mozart) and G2 (Ambience) were individually placed in an experimental chamber with red lighting, a metal floor, and walls for 8 min. At the 3rd, 4th, and 5th minutes, the mice were exposed to a sound stimulus of 72 dB frequency for 3s, followed by a foot shock of 0.75 mA for 2s. The chambers were cleaned with 70% ethanol before and after each treatment. The mice in group G3 (Control) did not undergo aversive training.^23^

### Extinction test

The extinction Test began 28 days after aversive training and lasted for five days (from the 82nd to the 86th day). The mice were placed in the same chamber used for aversive training and left for 10 min without foot shock. During this period, the freezing behavior of the animals was recorded. Freezing was defined as immobility of the head and body, with eyes fully open, and rapid breathing.^10^

Any behavior different from these was considered non-freezing behavior. The extinction test sessions were conducted over five consecutive days. All aversive training and extinction test sessions were recorded, stored, and transcribed using EthoLog software to analyze mouse behavior.^10^

### Recall test

Past 21 days since the last day of the the extinction test (107th day), a recall test was conducted in which the animals were re-exposed to the experimental chamber without receiving a shock, and their freezing time was scored at 2-min intervals.^23^

Freezing was defined as immobility of the head and body, with eyes fully open, and rapid breathing. Any behavior different from these was considered a non-freezing behavior.^10^

### Euthanasia

To achieve an anesthetic state before euthanasia by decapitation using a rodent guillotine, xylazine (2mg/kg) and ketamine (25mg/kg) were administered intramuscularly. After euthanasia, the animals were placed in red plastic bags and handed over to a company contracted by the Municipal Government of Itajubá, which was responsible for collecting waste and potentially contaminated materials.

### Statistical analysis

Statistical analyses were performed using IBM SPSS Statistics^®^ software (version 22). Raw data for freezing time (FT, in seconds) from aversive training for each animal were transformed into percentages using the following formula: (FT × 100)/480s, where 480s (8 min) was the duration of the session. The raw data for the FT (in seconds) from the extinction tests for each animal were transformed into percentages using the following formula: (FT × 100)/600s, where 600s (10 min) was the duration of the entire session. Likewise, raw data for the FT (in seconds) from the recall tests for each animal were transformed into percentages using the following formula: (FT × 100)/120s, where 120s (2 min) was the duration of the session.

All results are presented as mean percentage ± standard error of the mean (SEM). The comparison of data from the aversive training for the Mozart and Ambience Groups was conducted using Student’s *t*-test for independent samples (the Control Group did not undergo this training and was therefore excluded from the analyses). Data related to the 5 days of extinction tests (day 82 to day 86) were analyzed using repeated measures ANOVA, with the percentage of FT as the dependent variable, days as the within-subjects factor (or repeated measures), and groups as the between-subjects factor. As Mauchly’s test for sphericity was significant (χ^2^(9)=34.09; p<0.001), the Huynh-Feldt correction was applied (ε=0.827). Repeated contrast follow-up tests were used to investigate the temporal relationship over the days. Complementary (post-hoc) analyses were conducted using the Bonferroni test to evaluate the possible significant effects detected between groups (subjects). Data related to the recall session were analyzed using a one-way ANOVA, with groups as the independent variable, followed by the Bonferroni test if any significant effect was found. Statistical significance was set at p≤0.050.

## RESULTS

**Figure 2:**
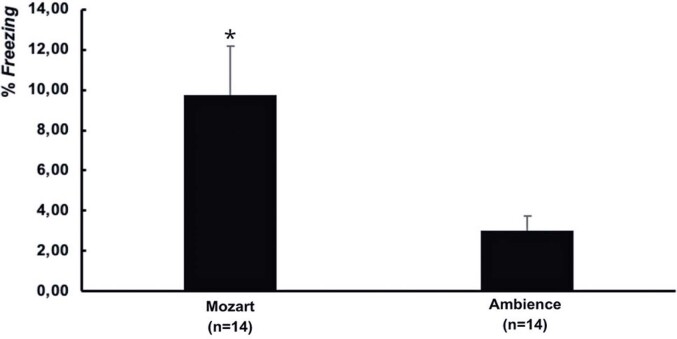
The average (standard error of the mean) freezing behavior time (%) during aversive training indicated a significant difference between the groups

There was a significant difference between the groups in terms of the number of aversive training sessions [t(26)=2.63, p=0.014; Figure 2]. The mean ± SEM percentage of freezing behavior for the Mozart Group (9.73% ± 2.44%) was statistically higher than that of the Ambience Group (3.03% ± 0.73%).

* t(26) = 2.63, p=0.014.

**Figure 3:**
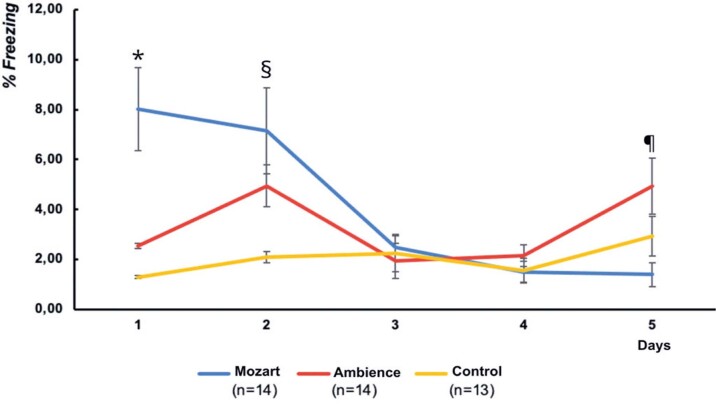
Average (standard error of the mean) freezing behavior time (%) over the 5 days of extinction sessions

Figure 3 shows the means (standard errors of the means) of the percentage of freezing time for the three groups over five days of fear extinction sessions. There was a significant main effect of day (F(3.31;125.72)=74.46; p<0.001). Follow-up contrasts between days 2 and 3 (F(1.38)=13.25; p=0.001) and days 4 *versus* 5 (F(1.38)=8.98; p=0.005) were significant, generally showing a decrease in freezing behavior over time.

*p<0.001 compared with the other two groups. §p=0.010, compared to the Control Group; ^¶^ p=0.014, compared to the Mozart Group.

There was a main effect of group [F(2.38)=4.01; p=0.026]. On day 1 [F(2.38)=13.10; p<0.001], the Mozart Group showed a significantly higher percentage of freezing behavior than the other two groups (all p≤0.001). On day 2 [F(2.38)=4.89; p=0.013], the Mozart Group showed a significantly higher percentage of freezing behavior than that of the Control Group (p=0.010). Finally, on day 5 [F(2.38)=4.58; p=0.016], the Mozart Group had a significantly lower percentage of freezing behavior than the Ambience Group (p=0.014).

There was also an interaction between days and groups [(F(6.62;125.72]=7.67; p<0.001), indicating that the fear extinction process differed among the groups over time. Follow-up contrasts revealed differences between groups on days 2 and 3 [F(2.38)=4.07; p=0.025] and between groups on days 4 and 5 [F(2.38)=3.54; p=0.039]. As shown in Figure 3, from the second to third day, the Mozart Group exhibited a more pronounced reduction in freezing behavior. Between days 4 and 5, while the Mozart Group maintained a low percentage of freezing behavior, the Ambience Group showed an increase in freezing behavior.

**Figure 4:**
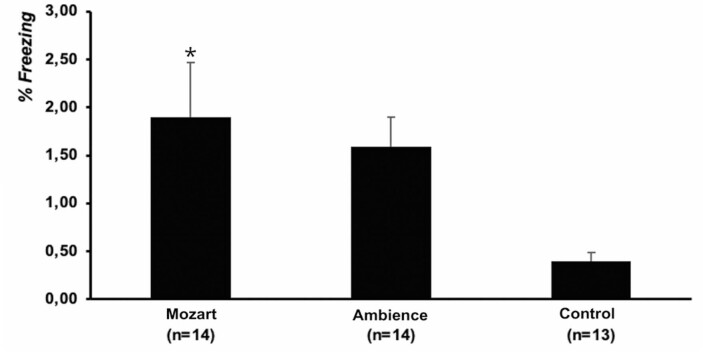
Average (standard error of the mean) freezing behavior time (%) during the recall session, indicating a significant difference between the groups [F(2.37)=4.21; p=0.022]

Finally, there was a significant difference between the groups in the recall test [F(2.37)=4.21; p=0.022]. The freezing behavior of the Mozart Group was significantly better than that of the Control Group (p=0.028; Figure 4).

*p=0.028, compared to the Control Group.

## DISCUSSION

To date, few studies have established a relationship between the extinction of contextual fear memory and exposure to Sonata K. 448. In the present study, from the first day of the extinction test, mice exposed to the sonata (Mozart Group) showed a statistically significant difference in freezing behavior. Moreover, during the test, the freezing behavior of these mice decreased faster than that of the other mice, albeit gradually. These findings indicate that music influences the extinction of contextual fearful memories.

Evidence has demonstrated an association between music exposure and memory formation through its effect on neural circuit modulation. For instance, animals exposed to Mozart’s Sonata K.448 show significant temporary improvements in tasks requiring spatial and temporal memory.^10,15^ There are also studies suggesting that prolonged exposure contributes to maintaining cognition and reducing memory decline with aging.^24^

However, the literature has scarcely addressed the effects of music exposure on memory extinction. This process results in the formation of a new memory that alters the neuronal circuitry by modulating the original memory. In other words, the behavior manifested in response to the stimuli was adapted. This resignification is of great adaptive value, preventing the persistence of behaviors or thoughts that no longer connect to reality.^5,9,10^

Consistent with our data, it is plausible to assume that exposure to Mozart’s Sonata K.448 before aversive training promotes neuroplasticity, thereby facilitating memory extinction. As physical exercise and sleep have shown benefits in the extinction of fear memory, music exposure, which has known effects on brain modulation, can enhance this process.^10,11,13,25-27^ This is due to the optimization of neurophysiological mechanisms and processes favoring adaptive learning, such as memory extinction.^13,15,25^ The repetition of conditioned behaviors is avoided by dissociating them from the context and aversive stimulus.^28-30^ Moreover, there is evidence of increased levels of brain-derived neurotrophic factor (BDNF), resulting in improvements in neurogenesis and synaptic plasticity due to musical exposure.^12,14,24^

In the recall test, the Mozart Group exhibited a longer freezing time than the Control Group (exposed only to ambient noise), demonstrating enhanced memory formation in mice exposed to music. This result resembles previous findings that during the memory consolidation period, exposure to a neutral context, similar to the conditioned context, leads to fear generalization of that neutral context.^5^In this sense, our results show that music exposure not only intensifies overall memory formation, including fear memory, but also makes the brain more prone to reformulating its response to aversive stimuli.

Therefore, this study encourages further research on the physiological mechanisms underlying the effects of music on memory extinction. Our limitations invite supplementary investigations that include more variables, such as female mice and diverse musical types, to enrich the outcomes and possibly widen their applicability in clinical settings.

## CONCLUSION

In agreement with these results, the increase in freezing time observed in male mice exposed to Mozart’s Sonata K.448 suggests that music affects memory formation. Complex auditory stimuli may enrich information retention. Thus, the elevation in freezing behavior suggests that the sonata has a significant influence on modulating the neural circuits involved in memory processing.

Nevertheless, male mice exposed to the sonata experienced a linear decline in their memory. The progressive decrease in freezing behavior demonstrates that the sonata may have initially reinforced information retention with a consecutive redefinition of the experience. This sequence of events was not observed in male mice from groups not exposed to the sonata (ambient and control), highlighting the impact of Mozart’s Sonata K.448 on cognitive functions.
